# Assessment of Biventricular Myocardial Function with 2-Dimensional Strain and Conventional Echocardiographic Parameters: A Comparative Analysis in Healthy Infants and Patients with Severe and Critical Pulmonary Stenosis

**DOI:** 10.3390/jpm12010057

**Published:** 2022-01-06

**Authors:** Liliana Gozar, Mihaela Iancu, Horea Gozar, Anca Sglimbea, Andreea Cerghit Paler, Dorottya Gabor-Miklosi, Rodica Toganel, Amalia Făgărășan, Diana Ramona Iurian, Daniela Toma

**Affiliations:** 1 Emergency Institute of Cardiovascular Diseases and Transplantation, 540139 Târgu-Mureș, Romania; lili_gozar@yahoo.com (L.G.); aisglimbea@gmail.com (A.S.); palerandreea@yahoo.com (A.C.P.); annadorka@yahoo.com (D.G.-M.); rodica.toganel@umfst.ro (R.T.); amalia.fagarasan@umfst.ro (A.F.); dianaiurian@yahoo.com (D.R.I.); daniela.toma@umfst.ro (D.T.); 2 Department of Pediatrics, “George Emil Palade” University of Medicine, Pharmacy, Science and Technology of Târgu-Mureș, 540139 Târgu-Mureș, Romania; 3 Department of Medical Informatics and Biostatistics, Faculty of Medicine, “Iuliu Hațieganu” University of Medicine and Pharmacy Cluj-Napoca, 400012 Cluj-Napoca, Romania; 4 Department of Pediatric Surgery, “George Emil Palade” University of Medicine, Pharmacy, Science and Technology of Târgu-Mureș, 540139 Târgu-Mureș, Romania; horea.gozar@umfst.ro

**Keywords:** pulmonary stenosis, speckle tracking, myocardial strain, infants, echocardiography

## Abstract

Our aim was to compare the global longitudinal and regional biventricular strain between infants with severe and critical pulmonary stenosis (PS), and controls; to compare pre- and post-procedural strain values in infants with severe and critical PS; and to assess the correlations between echocardiographic strain and conventional parameters. We conducted a retrospective single-center study. The comparisons of echocardiographic variables were performed using separate linear mixed models. The overall mean right ventricle (RV) regional strains measured before intervention in PS patients was significantly different when compared to the control group (*p* = 0.0324). We found a significant change in the left ventricle, RV, and inter-ventricular septum strain (IVS) values from basal to apical location (*p* < 0.05). IVS strain values showed a higher decrease in mean strain values from basal to apical in PS patients. There was no significant difference in means of baseline and post-interventional strain values in PS patients (*p* > 0.05). Following the strain analysis in patients with PS, we obtained statistically significant changes in the RV global-4-chamber longitudinal strain (RV4C). The RV4C, which quantifies the longitudinal strain to the entire RV, can be used in current clinical practice for the evaluation of RV function in infants with severe and critical PS. The longitudinal and segmental strain capture the pathological changes in the IVS, modifications that cannot be highlighted through a classical echocardiographic evaluation.

## 1. Introduction

Valvular pulmonary stenosis (PS), defined as an obstruction of blood flow at the level of the pulmonary valve, is a form of congenital heart disease, described in 0.6–0.8 out of 1000 live births. Critical valvular PS is the most severe form, characterized by complete obstruction of the right ventricular (RV) outflow tract, requiring maintenance of ductal patency through continuous prostaglandin infusion for the preservation of pulmonary circulation. Severe PS does not require maintenance of the arterial ductus patency, and is determined by a maximum instantaneous gradient across the pulmonary valve above 60 mmHg [1,2]. Percutaneous balloon valvuloplasty represents the treatment of election in newborns with critical valvular PS, and in infants with severe stenosis, the procedure being promoted by Kan et al. [3]. Studies found in the literature mostly focus on the residual gradient and on the recurrence rate of PS in children who have undergone an interventional procedure, respectively. RV decompression is first preceded by a considerable increase of afterload at the RV level. Transient left ventricular (LV) dysfunction after the interventional treatment of valvular PS has been described in some patients [4,5,6,7,8]. Often, routine echocardiographic examination is based on the determination of the following parameters: ejection fraction (EF), mitral (MAPSE) and tricuspid annular plane systolic excursion (TAPSE), and diastolic function. Also, classical echocardiography is limited in the evaluation of the RV function and in the differentiated evaluation of the interventricular septum and ventricular wall, respectively, as these aspects may represent key elements in the congenital heart disease. Lately, the usefulness and value of the speckle-tracking method in assessing ventricular function has been proven in a number of publications, and that of the LV peak global longitudinal strain (LVpGLS), respectively, which is considered a parameter of reference. The European Association for Cardiovascular Imaging (EACVI) and the American Society of Echocardiography (ASE) recommend the additional use of LVpGLS in the evaluation of LV EF [9]. However, the use of this method in the current clinical practice is limited [10,11]. This imaging technique enables a new perspective on the complexity of cardiac mechanics, being able to provide elements of early prognosis when compared to standard echocardiography, through the subtle changes highlighted by myocardial deformation measurements [12,13]. Current studies focus on aortic and mitral pathologies, whereas data on valvular diseases involving the right side of the heart are limited [12].

We aimed to assess the biventricular myocardial function in PS using conventional echocardiography and 2D-strain analysis by the following objectives: (1) comparisons of global longitudinal and segmental strains (measured pre- and post-interventional procedure) in PS patients to the control group; (2) comparison of global longitudinal and segmental strain pre- and post- interventional procedure in PS patients; (3) assessing the correlations between conventional parameters (such as MAPSE and TAPSE) and strain parameters.

## 2. Materials and Methods

The retrospective study was performed in the Children’s Cardiology Clinic from the Emergency Institute of Cardiovascular Diseases and Heart Transplantation in Targu Mures, Romania, for a period of 2 years, more precisely between August 2019 and September 2021. Infants with severe and critical PS, with an indication for interventional treatment, in whom the pre- and post-procedural echocardiographic evaluation performed at 24 h were available in the database, were included in the study. A control group of healthy infants was also formed.

Inclusion criteria: infants with ages between 0 and 12 months diagnosed with critical or severe valvular PS, with an indication for interventional therapy, in cases in which there was no need for the maintenance of ductal patency post-procedurally, with tripartite structure of the RV, complete pre- and post-procedural echocardiographic evaluation performed in the first 24 h.

Exclusion criteria: patients with other associated cardiac malformations, significant infundibular PS, severe pulmonary regurgitation, RV hypoplasia, RV pressure dependent coronary circulation proved by angiography, and patients in whom the available echocardiographic images were insufficient for speckle-tracking analysis, respectively.

Control group inclusion criteria: healthy infants referred to the cardiology department for the detection of an innocent systolic murmur, with no personal medical history, no cardiac or extracardiac pathology, appropriate linear and ponderal growth, with echocardiographic images available in the database.

All echocardiographic evaluations were performed using an Epiq 7 ultrasound machine. The echocardiographic parameters extracted from the database and found in each patient were the following: lateral MAPSE and TAPSE, LV EF-Teichholz, as well as the maximum transvalvular pulmonary gradient (PG). The two-dimensional acquisitions, recorded with a frequency (frame rate) of over 60 Hz and optimal quality, from an apical 4-chamber view and stored in Digital Imaging and Communication in Medicine (DICOM) format, were obtained from the database. Acquisitions were analyzed offline using Philips QLAB 15 software, and the LV autostrain and RV autostrain functions, respectively. The cardiac cycle was defined in M-mode by the movement (closing and opening, respectively) of the mitral valve, and was generated automatically by the software. Three endocardial points were chosen to mark the base of the septum, and the lateral and apical margins. The edge of the endocardium was automatically drawn, then verified and corrected manually.

**Figure 1:**
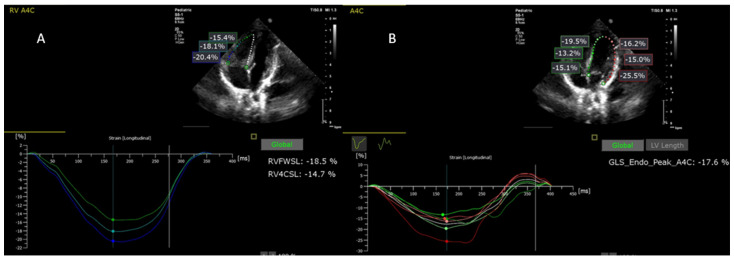
Biventricular speckle tracking analysis: (**A**) right ventricle speckle tracking analysis; RVFWSL—right ventricular free wall longitudinal peak systolic strain; RV4CSL—right ventricle four-chamber longitudinal strain; (**B**) left ventricle speckle tracking analysis; GLS_Endo_Peak_A4C (denoted by LV pGLS in our study)—peak global longitudinal strain of the left ventricle from apical four-chamber view.

Thus, the pre- and post-procedural images of the patients included in the study were analyzed, measuring both the pGLS and the biventricular segmental strain, the interventricular septum, and the walls being divided into three segments (LV Basal, LV Medial, LV apical, Inter V basal, Inter V Medial, Inter V Apical, RV Basal, RV Medial, RV Apical, RV free Wall, RV4C), and then their values were compared to the echocardiographic parameters measured in the control group (Table 1, Figure 1).

The speckle analysis was performed by a single evaluator, a pediatric cardiologist with experience in both classical echocardiography, and speckle tracking analysis.

## 3. Ethics

The study was approved by the ethics committees of the Institute of Cardiovascular Diseases and Transplantation, and “George Emil Palade” University of Medicine, Pharmacy, Science, and Technology of Targu Mures, 1276/25.02.2021.

## 4. Statistical Analysis

Demographic, anthropometric, clinical, and echocardiographic variables were summarized by frequencies (%), mean (SD = standard deviation), or median (IQR = interquartile interval). Differences concerning demographic, anthropometric, and clinical variables between the control and PS groups were examined by a Chi-squared test, Fisher’s exact test, Student-t test, or Mann–Whitney test. The distribution of continuous variables was checked for univariate normality using a Shapiro–Wilk test, normal Q-Q plot, skewness-kurtosis plot, or measures for the comparison of the fit between normal to empirical data distributions (such as Akaike information criteria (AIC) and Schwarz’s Bayesian information criteria (BIC)).

The comparisons of echocardiographic variables (conventional, regional, and longitudinal strain) were performed using separate linear mixed models. These models included a random intercept specific to subject_id. We studied the effect of group (PS versus controls) and the effect of segment on the variation of strain parameters, and they were entered as fixed effects in models, whereas age and gestational age were considered as covariates. We also studied the effect of group on the variation of segmental strain values by adding an interaction term, group × segment, in the previously studied models.

The relationship between conventional echocardiographic and speckle-tracking variables stratified by group type was investigated by Pearson’s correlation coefficient (R) or Spearman’s correlation coefficient (the latter being used in the case of data distributions that did not follow a normal distribution).

All statistical tests were two-sided with a significance level (α) chosen at 0.05. Statistical analysis was performed in R software, version 4.1.1 (R Foundation for Statistical Computing, Vienna, Austria).

## 5. Results

During the aforementioned period, 15 cases were identified in the database of our clinic, consisting of infants with valvular PS, in which percutaneous valvuloplasty was performed, two of them being excluded from the study due to suboptimal echocardiographic images, and one with hypoplastic RV. Thus, the analyzed group consisted of 12 infants, 5 of them being diagnosed with critical valvular PS, in whom all of the available pre- and post-procedural data were evaluated. As for the control group, it consisted of 50 healthy infants.

### 5.1. Description of Baseline Characteristics of PS Patients and Controls

Table 2 summarizes the baseline *(measured before interventional procedure)* characteristics of the PS patients and controls. The studied groups were similar regarding gender distribution (*p* = 0.330) and birth weight (*p* = 0.935), but there was a significant difference in age and gestational age distribution (*p* < 0.05), with patients with PS having smaller age values compared to controls (median (IQR): 4.5 (2, 14.75); range value of 2–24 weeks vs. 13 (8,16); range value of 3–26 weeks).

### 5.2. Comparison of PS Cases with Control Group in Terms of Baseline Regional and Global Longitudinal Strain

The group comparisons (PS patients versus controls) at each biventricular region (basal, medial, and apical) showed no significant group effect on baseline LV and inter-ventricular regional strain values, but a significant group effect was found regarding baseline RV regional strain measurements (Table 3). We found a significant change in LV, RV, and inter-ventricular septum strain values from basal to apical location (*p* < 0.05).

Patients with PS experienced a significant basal-to-apical difference (*p* = 0.0046) and basal-to-medial difference of the LV regional strain values (*p* < 0.0001), with the lowest value of strain at the medial location, and the greatest strain in the LV basal location (estimated marginal means: −12.8%, 95% CI: (−17.2, −8.27) for medial segment; −20.2%, 95% CI: (−24.7, −15.75) for apical region; and −31.6%, 95% CI: (−36.1, −27.13) for basal region). Similar significant differences were found in the control group (estimated marginal means: −16.4%, 95% CI: (−18.5, −14.22) for medial region; −14.6%, 95% CI: (−16.8, −12.5) for apical region; and −28.5%, 95% CI: (−30.6, −26.4) for basal region).

Concerning the RV strain values, we found a significant change from the basal-to-apical region (*p* = 0.026) and basal-to-medial region (*p* < 0.0001) in PS patients, with the smallest value of strain at the apical location, and the greatest strain at the basal location (estimated marginal means: −19.7%, 95% CI: (−23.2, −16.1) for medial region; −17.5%, 95% CI: (−21.1, −14.0) for apical region; and −23.6%, 95% CI: (−27.2, −20.0) for basal region). The pattern of change was the same for controls (estimated marginal means: −23.2%, 95% CI: (−24.9, −21.6) for medial region; −19.5%, 95% CI: (−21.2, −17.9) for apical region; and −28.5%, 95% CI: (−30.2, −26.8) for basal region).

Concerning the inter-ventricular regional strain, we found that patients with PS experienced only a significant basal-to-apical difference (*p* = 0.0002) (estimated marginal means: −16.4%, 95% CI: (−20.4, −12.4) for medial segment; −25.7%, 95% CI: (−29.7, −21.7) for apical segment; and −15.9%, 95% CI: (−12.9, −11.9) for basal segment). In the control group, there was a reversed pattern in strains compared to PS patients (the lowest mean value was found in the basal inter-ventricular region), with a significant basal-to-medial difference (estimated marginal means: −22.8%, 95% CI: (−24.7, −20.9) for medial segment; −20.9%, 95% CI: (−22.8, −19.0) for apical segment; and −18.0%, 95% CI: (−19.9, −16.1) for basal segment).

The interaction effect (group × medial location) on LV strain values had a marginal significance (*p* for interaction = 0.053), with the estimated marginal means showing a tendency towards having the greatest value of strain from the basal-to-LV medial region in PS patients when compared to controls (−18.86, 95% CI: (−27.81, −9.91) for PS group vs. −12.13, 95% CI: (−16.51, −7.74) for controls).

The results showed a significant interaction effect (group × apical location) on RV strain values and the inter-ventricular septum (*p* for interaction = 0.036 and *p* = 0.005, respectively). The RV regional strain values decreased in average in both groups, but a higher decrease in mean values from the basal-to-apical region was observed in controls in comparison to the PS group (−8.95, 95% CI: (−10.69, −7.21) for controls vs. −6.06, 95% CI: (−9.60, −2.51) for PS patients). Concerning the inter-ventricular strain values, the results showed a higher decrease in mean strain values from the basal-to-apical region in PS patients as opposed to controls (9.8, 95% CI: (3.61, 16.05) for PS group vs. 2.91, 95% CI: (−0.14, 5.96) for controls).

The group comparisons (PS patients versus controls) showed a significant group effect on RV4C strain values (*p* = 0.005), with PS patients having a smaller value of RV4C strain than controls (estimated marginal means: −16.5%, 95% CI: (−19.1, −13.9) in PS group vs. −20.8%, 95% CI: (−22.0, −19.5) in controls). We found no significant group effect in LVpGLS (*p* = 0.674) and RV free (*p* = 0.102).

### 5.3. Comparison of PS Cases with Control Group in Terms of Regional and Global Longitudinal Strains Measured after Interventional Procedure for the PS

The group comparisons (PS patients versus controls) at each region (basal, medial, and apical) showed no significant group effect on LV and inter-ventricular regional measurements of strain values after the interventional procedure for the PS, but a significant group effect was found on RV regional strain measurements (Table 4). We found a significant change in LV, RV, and inter-ventricular septum strain values from the basal-to-apical location (*p* < 0.05).

We found no significant difference in the mean value of global longitudinal strain (LVpGLS: *p* = 0.574; RV free: *p* = 0.642; RV4C: *p* = 0.106) between the two groups (estimated marginal means for LVpGLS: −18.7%, 95% CI: (−21.4, −15.9) in PS group vs. −19.6%, 95% CI: (−20.8, −18.3) in controls; RV free: −23.1%, 95% CI: (−26.6, −19.6) in PS group vs. −24.0%, 95% CI: (−25.6, −22.4) in controls; RV4C: −18.3%, 95% CI: (−21.0, −15.6) in PS group vs. −20.8%, 95% CI: (−22.0, −19.6) in controls).

### 5.4. Changes in Baseline and Post-Procedural Biventricular Regional Strains in PS Patients

We found no significant change in left ventricular, right ventricular, or inter-ventricular regional strains baseline and post-intervention in PS patients (*p* > 0.05) (Table 5).

### 5.5. Comparison of Longitudinal Strain and Conventional Ecocardiographic Parameters Measured before and after Interventionin in PS Patients

There was a significant change in TAPSE mean (*p* = 0.015), and PG (*p* < 0.0001), but no significant change in longitudinal strain value mean (*p* > 0.05) in PS patients (Table 6).

### 5.6. Correlations between Global Longitudinal Strain Variables and Conventional Ecocardiographic Parameters by Group Type

**Figure 2:**
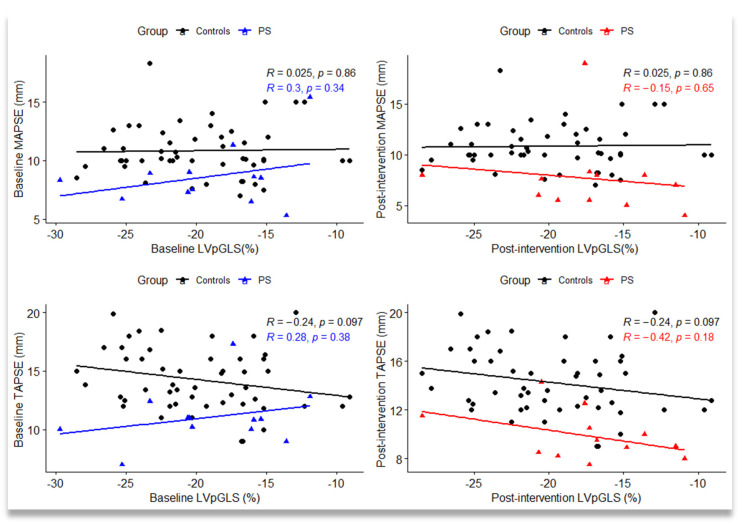
Scatterplot between left ventricle peak global longitudinal strain (LVpGLS), mitral annular plane systolic excursion (MAPSE), and tricuspid annular plane systolic excursion (TAPSE) measured baseline and after intervention in the control group and pulmonary stenosis (PS) group; R = Pearson’s correlation coefficient; statistical significance: *p* < 0.05.

We noticed a negative linear correlation between LVpGLS and TAPSE in the control group (r = −0.24), with a tendency towards statistical significance (*p* = 0.097). In the PS group, we found no significant correlation between LVpGLS and TAPSE (*p* = 0.38) before intervention, but after intervention, the correlation had the same direction (r = −0.42) as in the control group (Figure 2).

**Figure 3:**
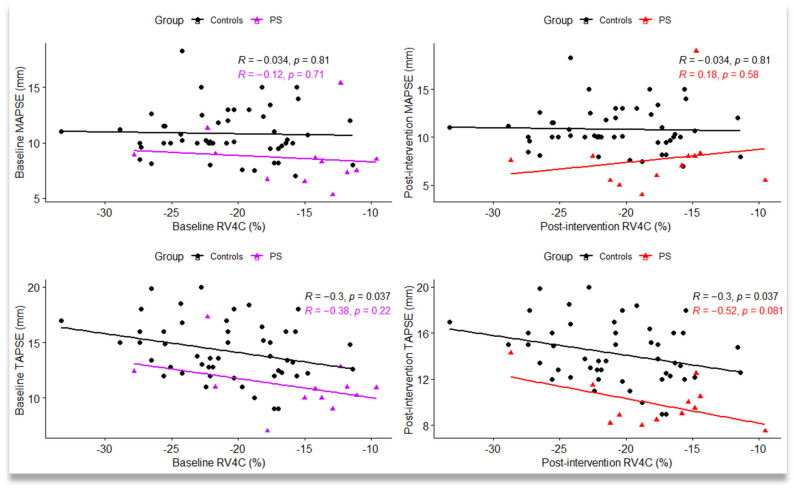
Scatterplot between right ventricle four-chamber strain (RV4C), mitral annular plane systolic excursion (MAPSE), and tricuspid annular plane systolic excursion (TAPSE) measured in the control group and pulmonary stenosis (PS) group (baseline and after intervention); R = Pearson’s correlation coefficient; statistical significance: *p* < 0.05.

There was a significant negative linear correlation between RV4C and TAPSE in the control group (r = −0.30, *p* = 0.037). In PS group before intervention, we also noticed a negative linear correlation between baseline RV4C and TAPSE (r = −0.38), but without statistical significance, whereas after the intervention, the strength of linear correlation between RV4C and TAPSE was higher (r = −0.52), with a tendency towards statistical significance (*p* = 0.081) (Figure 3).

We also calculated the percent change relative to baseline of conventional and strain longitudinal parameters in PS patients, and we found a positive correlation between the percent change in LVpGLS and MAPSE (ρ =0.58), with a marginal significance (*p* = 0.052). We noticed a negative correlation between the percent change in RV free and RV4C with TAPSE (ρ = −0.41 and ρ = −0.36), but without statistical significance (Table 7).

## 6. Discussion

Understanding and quantifying ventricular function is of primordial importance in the treatment of congenital heart disease associated with heart failure. Petiijean et al. have made a complex analysis of the global longitudinal and segmental deformation of the heart. At RV level, the dynamic analysis of the longitudinal contraction shows that the displacement increases at the basal and medio-ventricular levels, but not at the apex, whereas the longitudinal deformation increases from the base to the apex [14]. The architecture of the LV myocardium is complex. The subendocardial and subepicardial fibers are directed longitudinally, whereas those in the medial portion are positioned circumferentially. This particular arrangement of the myocardial fibers leads to complex and inhomogeneous patterns of contraction [14]. The magnitude of the strain is dependent on both the pre- and afterload of the ventricle, and the intrinsic tension of the myocardial fibers [15]. The ventriculo–ventricular interaction and the position of the interventricular septum also play an essential role in cardiac hemodynamics.

This study aims to investigate the dynamics of the following parameters: MAPSE, TAPSE, and longitudinal and segmental strain values in the light of RV pathology, through the observation of infants with critical and severe PS. Studies previously published in the literature regarding this pathology have focused mainly on the long-term outcome of patients who have undergone percutaneous pulmonary balloon valvuloplasty, with the documentation of residual lesions and the recurrence of stenosis [16]. Given the current state of knowledge, the assessment of cardiac function using two-dimensional speckle tracking in children is conditioned by the knowledge of reference range values [17]. It is also known that strain values vary depending on the analysis software [18]. In light of the aforementioned data, we considered a comparative analysis with a control group necessary.

In our study, in the case of patients with PS, the segmental strain analysis showed higher values at the LV and RV level; more precisely, at the level of the basal segments when compared to the medial and apical segments, with the differences between segmental strain values being statistically significant: basal-to-apical and basal-to-medial gradient. Similar changes were observed in the control group.

In the PS group, we found a significant difference for the interventricular septum regional strains between the basal and apical segments (the lowest strain value being found in the basal segment, and the highest in the medial segment), whereas in the control group, we noticed a reversed pattern in strain (the lowest strain value in the basal segment, but the highest in the apical segment). Our results confirm reported results from Schubert et al. [19]: the decrease of the gradient from the base to the apex in newborns; the tendency towards increased strain values at the level of the basal segments being specific to the fetal period; mentioning the fact that this gradient difference between basal-, medial-, and apical segments, respectively, is maintained postnatally. These results illustrated through the speckle-tracking technique are explained by the multiple and complex processes of cardiopulmonary transition between fetal and neonatal life [18,19]. In a meta-analysis consisting of 2325 children, with ages between 0–21 years, Levy et al. show that there is a statistically significant difference between the basal and apical segments, and apical-to-basal gradient (highest to lower) in the LV free wall [17]. Moreover, Jashari et al. concluded that the basal-to-apical LV gradient appears with age, and becomes statistically significant in the age groups of 5–9 and 10–14 years, respectively [18,19].

In our study, the analysis of the longitudinal strain highlights the RV4C parameter as being significantly lower in the group of patients with PS before the interventional procedure. RV4C quantifies the RV longitudinal strain including the interventricular septum. Existing data in the literature correlate the longitudinal systolic peak with global hemodynamic changes [20]. Following the longitudinal and segmental strain analysis in patients with PS, we obtained statistically significant changes in the RV4C, whereas the RV free wall parameter was without statistical significance. The segmental analysis performed at the level of the intraventricular septum revealed differences in basal-to-apical gradients between the two groups (reverse pattern in the control group), whereas results obtained at the level of the ventricular walls showed no differences. These combined elements direct us towards the idea that the interventricular septum is modified by right ventricular pathology, with the two ventricles being in a direct interaction through the septum. Thus, an increase in the volume of one of the ventricles may induce changes in volume and pressure values in the other. In the case of a heart with an intact pericardium, the ventricular pressure–volume relationship is deviated to the left by the increase in pressure in the contralateral ventricle, with a pressure transmission from the LV to the RV of approx. 30%, and from the RV to the LV of >50% [21]. Subtle changes in strain values may be useful for a more accurate analysis of interventricular interaction, prognosis, and management of patients undergoing percutaneous balloon valvuloplasty [7].

Performing the same analysis after the interventional procedure shows the maintenance of the differences between the basal and apical segments, with the following remarks: in the case of the LV, the decrease of strain values between basal and medial segments is significant in the PS group, whereas in the case of the RV, the difference between basal-to-apical segments is higher in the control group. Changes in this gradient suggest modifications in segmental deformation that occur at the cardiac level secondary to changes in ventricular afterload.

Strain parameters in the PS group were analyzed before and immediately after the interventional procedure, thus we were able to notice that strain parameters examined in the first 24 h post-procedurally do not capture the effect of the intervention, as differences may be perceived only through classical echocardiographic evaluation due to the decrease of the transvalvular gradient, and by the improvement of the TAPSE. Considering the fact that PS patients were hemodynamically stable, both pre- and post-procedurally, changes in longitudinal and segmental deformation were also minimal. On the other hand, linear correlations between longitudinal strain, TAPSE, and MAPSE, respectively, present some interesting aspects. A negative linear correlation was obtained between parameters LVpGLS and TAPSE and the strain parameter, respectively, represented by RV4C and TAPSE, both in the control and the PS group. This negative correlation indicates that these parameters change in the same direction.

Furthermore, LVpGLS and MAPSE adjusted for baseline values vary in the same direction (Table 6). The negative correlation between TAPSE and RV free wall has been described in adult patients with RV dysfunction in the context of secondary pulmonary hypertension by Kemal et al. [22]. Kempny et al. capture similar variability between MAPSE, LVpGLS, and TAPSE, respectively, and RV longitudinal strain in adults with RV dysfunction after surgical correction of Fallot’s tetralogy [23]. In light of these data, the combination of classical echocardiography parameters with the analysis of global and segmental deformation brings a plus in the evaluation of ventricular function in patients with RV pathology.

## 7. Limitations

The main limitation of this study is represented by its retrospective nature, an aspect that does not allow the optimization of the acquisitions for speckle tracking. In order to minimize this drawback, we excluded from the study group the cases that did not have good echocardiographic images, with a frequency below 60 Hz. Also, this study allowed us to evaluate only a smaller number of classical echocardiographic parameters, more precisely TAPSE and MAPSE, as these were available in the database for all patients. However, we considered these findings to be important for the clinical practice of pediatric cardiology, as these parameters are part of the routine echocardiographic evaluations, and are extremely easy to determine.

Because the current study contains data from a single center, the only center in the country that handles pediatric cardiology, there are some limitations. A limitation is given by the inclusion of a relatively small number of cases in the studied period (seven cases with severe stenosis, five cases with critical PS, respectively). It would have been beneficial to perform a differential analysis for each pathology; therefore, an extension of the study over a larger period is recommended. Although the strong point of the present study was the analysis of strain parameters between PS cases and controls taking into account the effect of some relevant factors (subjects’ age and within-subject variability), future studies are needed to confirm the utility in clinical practice, and the generalizability of the study findings. Because, in the current study, the evaluation of strain biventricular values was performed within 24 h from intervention for PS, in order to prove that longitudinal strain parameters can detect the subtle myocardial changes after intervention, future studies with multiple time points of strain measurements can fully evaluate the generalizability of significant changes in myocardial function before and after intervention in PS patients.

## 8. Conclusions

Results of the current study showed that the RV4C parameter allows the quantification of the longitudinal strain of the entire RV, and, due to its easy determination, it may be used in clinical practice in order to assess the RV function in infants with severe and critical PS. Longitudinal and segmental strains capture the pathological changes in the interventricular septum, modifications that cannot be highlighted through a classical echocardiographic evaluation. Speckle tracking analysis, alongside classical echocardiographic parameters, represent a useful technique in assessing cardiac function in infants with congenital heart disease.

## Figures and Tables

**Table 1:** Description of measures: speckle tracking and conventional variables.

Variables	Description
RV Basal	The basal segment of the right ventricle lateral wall
RV Medial	The medial segment of the right ventricle lateral wall
RV Apical	The apical segment of the right ventricle lateral wall
RV free wall	Longitudinal strain of the right ventricle free wall
RV4C	Global longitudinal strain of the right ventricle including interventricular septum
LV Basal	The basal segment of the left ventricle lateral wall
LV Medial	The medial segment of the left ventricle lateral wall
LV Apical	The apical segment of the left ventricle lateral wall
Inter V Basal	The basal segment of the inter-ventricular septum
Inter V Medial	The medial segment of the inter-ventricular septum
Inter V Apical	The apical segment of the inter-ventricular septum
LV pGLS	Peak global longitudinal strain of the left ventricle
GP max	Anterograde peak pressure gradient measured transpulmonary
EF M mode	Ejection Fraction
TAPSE	Tricuspid annular plane systolic excursion
MAPSE	Mitral annular plane systolic excursion

**Table 2:** Baseline characteristics of control and PS groups.

	Control Group (*n*_1_ = 50)	PS Group (*n*_2_ = 12)	*p*-Value
Gestational age (weeks) (1)	38.24 ± 0.77	38.92 ± 0.79	0.0086 *
Age (weeks) (2)	13 (8, 16)	4.5 (2, 14.75)	0.027 *
Gender (male) (3)	22 (44)	3 (25)	0.330
Body surface area (m^2^) (1)	0.27 ± 0.03	0.26 ± 0.06	0.766
SaO2 (%) (2)	99 (99, 100)	94.5 (85, 98.25)	<0.0001 *
Systolic blood pressure (mmHg) (2)	75.5 (70.5, 85)	83 (75.5, 93)	<0.041 *
Diastolic blood pressure (mmHg) (2)	51.4 (46.25, 55)	49.5 (39.5, 60)	0.604
Heart rate (bpm) (1)	128.5 ± 9.20	133.50 ± 18.41	0.380
Birth weight (g) (1)	3285 ± 541.85	3300 ± 657.99	0.935
Apgar score at 1-minute (2)	10 (9, 10)	9 (8, 10)	0.025 *
Apgar score at 5-minute (2)	10 (10, 10)	9.5 (9, 10)	0.0006 *
C-section (3)	11 (22)	5 (41.67)	0.268
Pathological pregnancy (3)	4 (8)	5 (41.67)	0.010 *
Genetic syndrome (3)	NA	4 (33.33)	NA
PS requiring prostaglandin infusion (3)	NA	5 (41.67)	NA
Ecocardiografic parameters			
EF	71.70 ± 6.34	70.33 ± 10.07	0.661
MAPSE	10.82 ± 2.24	8.61 ± 2.62	0.004 *
TAPSE	14.25 ± 2.65	11.03 ± 2.48	0.0003 *

Data are presented as (1) arithmetic mean ± standard deviation; (2) median (IQR), IQR = interquartile range defined by lower (Q1) and upper quartile (Q3); or (3) number of cases (n) and relative frequencies (%); NA = not available; * statistical significance: *p*-value < 0.05; baseline = measured before interventional procedure in PS patients; SaO2 (%) = oxygen saturation in the arterial blood; Apgar score at 1-minute = Apgar score at 1 min after birth; Apgar score at 5-minute = Apgar score at 5 min after birth; C-section = birth by cesarean section; PS requiring prostaglandin infusion = patient with severe pulmonary stenosis requiring treatment soon after birth with prostaglandin infusion; EF = ejection fraction M mode; MAPSE = mitral annular plane systolic excursion; TAPSE = tricuspid annular plane systolic excursion.

**Table 3:** Baseline regional biventricular strain variables measured in PS patients and controls.

Effects	Control Group	PS Group	Adjusted Difference between Groups ^(a)^		Mixed Models Analysis, *p*-Values ^(c)^		
	Mean ± SD	Mean ± SD	Estimated Regression Parameter [95% CI]	*p*-Value ^(b)^	PS Group (vs. Control)	Medial Location (Vs. Basal)	Apical Location (vs. Basal)
Location of the left ventricle 2D strain measurements					0.223	<0.0001 *	<0.0001 *
Basal	−28.83 ± 9.70	−30.22 ± 11.87	3.12 (−4.22, 10.45)	0.841			
Medial	−16.70 ± 4.21	−11.37± 4.26	−3.62 (−10.95, 3.72)	0.715			
Apical	−14.95 ± 7.67	−18.85 ± 8.04	5.62 (−1.72, 12.93)	0.239			
Location of the right ventricle 2D strain measurements					0.0324 *	<0.0001 *	<0.0001 *
Basal	−28.68 ± 6.72	−22.75 ± 8.69	−4.82 (−11.27, 1.64)	0.261			
Medial	−23.43 ± 6.19	−18.86 ± 6.88	−3.46 (−9.91, 2.99)	0.628			
Apical	−18.98 ± 7.78	−16.69 ± 5.99	−1.18 (−7.63, 5.28)	0.994			
Location of the inter-ventricular septum 2D strain measurements					0.3589	<0.0001 *	0.0066 *
Basal	−18.17 ± 5.36	−15.10 ± 7.25	−2.08 (−8.63, 4.46)	0.941			
Medial	−22.96 ± 4.73	−15.59 ± 7.02	−6.39 (−12.93, 0.16)	0.060			
Apical	−21.08 ± 8.19	−24.92 ± 10.70	4.83 (−1.71, 11.38)	0.275			

^(a)^ adjusted for age and gestational age (weeks); 95% CI = 95% confidence level; SD = standard deviation; ^(b)^ *p*-values obtained from post-hoc pairwise comparisons based on Tukey’s test; ^(c)^ overall *p*-values for each fixed-effect term of linear mixed model; * significant result (*p*-value < 0.05).

**Table 4:** Comparison of post-procedural biventricular regional strains in PS patients with controls.

Effects	Control Group	PS Group	Adjusted Difference between Groups ^(a)^		Mixed Models Analysis, *p*-Values ^(c)^		
	Mean ± SD	Mean ± SD	Estimated Regression Parameter [95% CI]	*p*-Value ^(b)^	PS Group (vs. Control)	Medial Location (vs. Basal)	Apical Location (vs. Basal)
Location of the left ventricle 2D strain measurements					0.539	<0.0001 *	<0.0001 *
Basal	−28.83 ± 9.70	−25.33± 15.51	−1.65 (−9.36, 6.07)	0.989			
Medial	−16.70 ± 4.21	−14.37± 5.88	−0.49 (−8.20, 7.23)	1.000			
Apical	−14.95 ± 7.67	−17.52 ± 7.56	4.42 (−3.29, 12.14)	0.565			
Location of the right ventricle 2D strain measurements					0.0141 *	<0.0001 *	<0.0001 *
Basal	−28.68 ± 6.72	−22.89 ± 6.58	−5.25 (−11.35, 0.86)	0.133			
Medial	−23.43 ± 6.19	−22.42 ± 6.79	−0.47 (−6.57, 5.63)	0.999			
Apical	−18.98 ± 7.78	−21.22 ± 6.71	2.04 (−4.07, 8.14)	0.924			
Location of the inter-ventricular septum 2D strain measurements					0.111	<0.0001 *	0.0082 *
Basal	−18.17 ± 5.36	−13.58 ± 5.07	−3.43 (−9.60, 2.75)	0.598			
Medial	−22.96 ± 4.73	−15.59 ± 6.16	−6.20 (−12.38, −0.03)	0.048 *			
Apical	−21.08 ± 8.19	−20.79 ± 10.13	0.88 (−5.29, 7.06)	0.998			

^(a)^ adjusted for age and gestational age (weeks); 95% CI = 95% confidence level; SD = standard deviation; ^(b)^ *p*-values obtained from post-hoc pairwise comparisons based on Tukey’s test; ^(c)^ overall *p*-values for each fixed-effect term of linear mixed model; * significant result (*p*-value < 0.05).

**Table 5:** Comparison of baseline and post-intervention biventricular regional strains in PS patients.

Effects	Baseline (T0)	Post-Intervention (T1)		*p*-Values for Main Effects		
	Estimated Marginal Means [95% CI]	Estimated Marginal Means [95% CI]	*p*-Value	Time T1 vs. T0	Medial Location (vs. Basal)	Apical Location (vs. Basal)
Location of the left ventricle 2D strain measurements				0.206	<0.0001 *	0.004 *
Basal	−30.2 (−35.6, −24.82]	−25.3 (−30.7, −19.93)	0.795			
Medial	−11.4 (−16.8, −5.96]	−14.4 (−19.8, −8.96)	0.969			
Apical	−18.9 (−24.3, −13.45)	−17.5 (−22.9, −12.12)	0.999			
Location of the right ventricle 2D strain measurements				0.946	0.065	0.005 *
Basal	−22.8 (−26.4, −19.1)	−22.9 (−26.5, −19.2)	1.000			
Medial	−18.9 (−22.5, −15.2)	−22.4 (−26.1, −18.8)	0.525			
Apical	−16.7 (−20.3, −13.00)	−21.2 (−24.9, −17.6)	0.259			
Location of the inter-ventricular septum 2D strain measurements				0.591	0.862	0.010 *
Basal	−15.1 (−19.6, −10.6)	−13.6 (−18.1, −9.1)	0.994			
Medial	−15.6 (−20.1, −11.08)	−15.6 (−20.1, −11.08)	1.000			
Apical	−24.9 (−29.4, −20.4)	−20.8 (−25.3, −16.28)	0.688			

Baseline = pre-intervention; marginal means were estimated from the linear mixed model adjusted for age and gestational age, and having patients as random effec; * significant result (*p*-value < 0.05).

**Table 6:** Changes in longitudinal strain and conventional echocardiographic parameters in PS patients.

Effects	Baseline (T0)	Post-Intervention (T1)	*p*-Value
	Estimated Marginal Means (95% CI)	Estimated Marginal Means (95% CI)	
LVpGLS (%)	−19.2 (−21.8, −16.5)	−17.4 (−20.0, −14.8)	0.337
RVfree (%)	−19.9 (−23.4, −16.3)	−22.7 (−26.2, −19.1)	0.252
RV4C (%)	−15.8 (−18.4, −13.3)	−17.8 (−20.3, −15.3)	0.195
EF (%)	70.3 (64.8, 75.8)	62.2 (56.7, 67.8)	0.039 *
PG	108.0 (90.8, 125.2)	37.2 (20.0, 54.5)	<0.0001 *
TAPSE	11.03 (9.81, 12.3)	9.87 (8.64, 11.1)	0.015 *
MAPSE	8.61 (6.34, 10.9)	7.66 (5.39, 9.93)	0.106

Marginal means were estimated from the linear model adjusted for age and gestational age. LVpGLS: left ventricle peak global longitudinal strain; RV: right ventricle; RV4C: right ventricle four-chamber strain; EF: ejection fraction; PG: maximum transvalvular pulmonary gradient; TAPSE: tricuspid annular plane systolic excursion; MAPSE: mitral annular plane systolic excursion; * significant result (*p*-value < 0.05).

**Table 7:** Correlations between the percent change relative to baseline in longitudinal strain variables and classical echocardiographic parameters in PS patients.

	∆LVpGLS (%)	∆Rvfree (%)	∆RV4C (%)	∆EF (%)	ΔTAPSE (%)	∆MAPSE (%)
∆LVpGLS (%)	−15.04 (−29.37, 23.88)	−0.13 (0.683)	−0.03 (0.939)	0.31 (0.343)	0.19 (0.558)	0.58 (0.052)
∆RVfree wall (%)		21.91 (−3.62, 38.39)	0.91 (<0.0001)	−0.08 (0.800)	−0.41 (0.193)	−0.03 (0.921)
∆RV4C (%)			16.58 (1.04, 37.61)	0.05 (0.886)	−0.35 (0.266)	−0.16 (0.619)
∆EF (%)				−8.09 (−15.87, −2.55)	0.41 (0.185)	−0.16 (0.619)
∆TAPSE (%)					−8.15 (−17.36, −4.89)	0.47 (0.128)
∆MAPSE (%)						−9.84 (−33.05, −6.56)

∆ = percent change from baseline to post-intervention in classical and longitudinal strain parameters (post-baseline)/baseline × 100); medians (IQR) for percent change were provided on the diagonal of matrix, whereas Spearman’s correlation coefficients (*p*-values) were presented on the upper triangular part of matrix; statistically significant result: *p* < 0.05; LVpGLS: left ventricle peak global longitudinal strain; RV: right ventricle; RV4C: right ventricle four-chamber strain; EF: ejection fraction; TAPSE: tricuspid annular plane systolic excursion; MAPSE: mitral annular plane systolic excursion.

## Data Availability

The raw data presented in this study can be obtained upon reasonable request addressed to Liliana Gozar (lili_gozar@yahoo.com).
